# Efficient dealkalization of red mud and recovery of valuable metals by a sulfur-oxidizing bacterium

**DOI:** 10.3389/fmicb.2022.973568

**Published:** 2022-08-29

**Authors:** Duo-rui Zhang, Hong-rui Chen, Jin-lan Xia, Zhen-yuan Nie, Rui-Yong Zhang, Eva Pakostova

**Affiliations:** ^1^School of Minerals Processing and Bioengineering, Central South University, Changsha, China; ^2^Key Lab of Biometallurgy of Ministry of Education of China, Central South University, Changsha, China; ^3^Key Laboratory of Marine Environmental Corrosion and Bio-fouling, Institute of Oceanology, Chinese Academy of Sciences, Qingdao, China; ^4^Open Studio for Marine Corrosion and Protection, Pilot National Laboratory for Marine Science and Technology (Qingdao), Qingdao, China; ^5^Centre for Sport, Exercise and Life Sciences, Coventry University, Coventry, United Kingdom

**Keywords:** red mud (bauxite residue), bioleaching, dealkalization, metal recovery, sulfur bio-oxidation

## Abstract

Red mud (RM) is a highly alkaline polymetallic waste generated *via* the Bayer process during alumina production. It contains metals that are critical for a sustainable development of modern society. Due to a shortage of global resources of many metals, efficient large-scale processing of RM has been receiving increasing attention from both researchers and industry. This study investigated the solubilization of metals from RM, together with RM dealkalization, *via* sulfur (S^0^) oxidation catalyzed by the moderately thermophilic bacterium *Sulfobacillus thermosulfidooxidans*. Optimization of the bioleaching process was conducted in shake flasks and 5-L bioreactors, with varying S^0^:RM mass ratios and aeration rates. The ICP analysis was used to monitor the concentrations of dissolved elements from RM, and solid residues were analyzed for surface morphology, phase composition, and Na distribution using the SEM, XRD, and STXM techniques, respectively. The results show that highest metal recoveries (89% of Al, 84% of Ce, and 91% of Y) were achieved with the S^0^:RM mass ratio of 2:1 and aeration rate of 1 L/min. Additionally, effective dealkalization of RM was achieved under the above conditions, based on the high rates (>95%) of Na, K, and Ca dissolution. This study proves the feasibility of using bacterially catalyzed S^0^ oxidation to simultaneously dealkalize RM and efficiently extract valuable metals from the amassing industrial waste.

## Introduction

Red mud (RM) is a solid by-product generated *via* the caustic digestion of bauxite ores (i.e., the Bayer process) during the production of alumina (Khairul et al., 2019), which is usually stored in large quantities in storage tanks or in the open air through dry or semi-dry methods (Power et al., 2011; Xue et al., 2016). Approximately 4 billion tons of RM are stored worldwide, and the amount is increasing with the rate of 120 million tons per annum (Wang et al., 2019; Khanna et al., 2022). RM presents a potential hazard to groundwater, surface waters, soils, as well as ocean ecosystems, due to its high alkalinity (caused by an elevated Na content) and high concentrations of potentially toxic metals (Wang et al., 2019; Chao et al., 2022). In addition, due to its fine particle size, RM can cause dust hazards, affecting the surrounding and downwind areas (Klauber et al., 2016). Serious environmental and safety issues due to the generation, storage and disposal of this hazardous waste have previously been reported in the mining and metallurgy sectors (Power et al., 2011).

Besides the elevated content of potentially toxic metals in RM, its high alkalinity (usually pH > 10) poses an additional challenge for RM recycling. For example, the content of Na must be <1% before RM is suitable for the production of building materials (Kang and Kwon, 2017), and RM can be used as a soil aggregate only when its pH is neutral to mildly alkaline (He et al., 2012). Therefore, reducing the alkalinity of RM is crucial prior to certain applications. Currently available methods for RM dealkalization include water leaching (Zhu et al., 2015), acid neutralization (Johnston et al., 2010), mild hydro-chemical processes (Zhong et al., 2009), and wet carbonization (Li et al., 2016). However, the currently available methods have several disadvantages. For example, wet carbonization is costly (Wang et al., 2018), and water leaching can be time-consuming and may produce large amounts of alkaline liquid wastes (Löser et al., 2007). Bioleaching is generally accepted as an economical and environment-friendly approach (Qu and Lian, 2013), and bioleaching-based technologies could serve as a suitable alternative to the above RM treatment methods. To date, no reports have been published on dealkalization of RM and recovery of metals using acidophilic sulfur- and/or iron-oxidizing prokaryotes.

RM often contains refractory phases of Fe (mainly of hematite), Si (quartz), Al, Ca, K, Ni, Zn, Cu, and others (Klauber et al., 2011; Agrawal and Dhawan, 2021). Besides, RM is an attractive source of rare earth elements (REEs) such as Gd, Ce, Sc, Y, and Nd (Abhilash et al., 2014; Liu and Naidu, 2014). Chemical extraction of REEs from RM has been performed using H_2_SO_4_, HCl, HNO_3_, and SO_2_ gas (Abhilash and Meshram, 2019), and attempts have also been made to selectively extract metals such as Ti (Deng et al., 2017). The reported methods for metal extraction from RM include the following: acid leaching (Agatzini-Leonardou et al., 2008; Pepper et al., 2016; Kong et al., 2017; Li et al., 2021; Lallemand et al., 2022), roasting combined with water leaching (Zhu et al., 2015; Borra et al., 2016), bioleaching using fungi (Qu and Lian, 2013; Vakilchap et al., 2016; Pedram et al., 2020; Ilyas et al., 2021), chemoorganotrophic bacteria (Abhilash et al., 2021; Kiskira et al., 2021), and microalgae (CíŽková et al., 2019). Also, a combination of washing and electrodialysis has been used to treat RM and extract metals, respectively (Zhang et al., 2019).

Hydrometallurgy uses strong acids (and/or other harsh chemicals) to solubilize metals, while bioleaching with acidophilic chemolithotrophs uses biogenic H_2_SO_4_ generated *via* sulfur (S^0^) bio-oxidation (Brierley and Brierley, 2013; Marrero et al., 2015; Pakostova and Johnson, 2019; Zhang et al., 2020). Bioleaching of metals from RM represents a suitable alternative to the conventional approaches, minimizing the usage of concentrated acids and generation of potentially hazardous wastes, while simultaneously reducing the negative impacts of RM on the environment and human health.

In this work, we investigated simultaneous dealkalization of RM and solubilization of valuable metals *via* S^0^ oxidation by the moderately thermophilic acidophile *Sulfobacillus (Sb.) thermosulfidooxidans*. The bioleaching tests were conducted using two process designs (shake flasks and 5-L bioreactors). The main objective of this study was to prove the feasibility of using bioleaching as an alternative to conventional techniques to efficiently recover valuable metals from RM.

## Materials and methods

### Red mud and elemental sulfur

RM used in this study was generated *via* the Bayer process by the Aluminum Corporation of China, Jiaozuo, China, and S^0^ was obtained from School of Minerals Processing and Bioengineering, Central South University, Changsha, China. The pH value of the original RM was 10.82. The elemental composition of RM analyzed by X-ray fluorescence spectroscopy (XRF) was (in wt.%): Al, 12.5; Si, 11.2; Fe, 10.5; Ca, 13.1; Na, 6.5; Ti, 3.1; K, 2.3; O, 38.9; Ce, 0.05; Y, 0.01, and others, 1.84. The morphology of S^0^ and RM was characterized by scanning electron microscopy (SEM) (Supplementary Figure S1). The surface of S^0^ was smooth with particle size noticeably larger than that of RM. The surface of RM composed of sheet-like structures scattered among amorphous mass. The X-ray diffraction (XRD) analysis of primary phases (Supplementary Figure S2) indicated that the intact RM comprised mainly katoite, cancrinite, hematite, diaspore, and calcite, with minor amounts of muscovite, perovskite, and kaolinite.

### Bacterial strain and cultivation conditions

The moderately thermophilic bacterium *Sb. thermosulfidooxidans* strain YN-22 (GenBank Accession Number: DQ650351) used in this study is maintained at the Key Laboratory of Biometallurgy of the Ministry of Education of China, Changsha, China. *Sb. thermosulfidooxidans* is an acidophile (with a pH optimum of 1.9–2.4; Karavaiko et al., 1990) that is often abundant in low-pH, sulfur-rich environments, showing a high sulfur oxidation activity at elevated temperatures (Dopson and Lindström, 2004). Additionally, the strain YN-22 has a high tolerance for potentially toxic metals and has been shown to be able to oxidize sulfur even under relatively high pH conditions (up to 4.5; Zhang et al., 2021). The basal salts medium (BSM) for cultivation comprised (in g/L): (NH_4_)_2_SO_4_, 3.0; MgSO_4_, 0.5; K_2_HPO_4_, 0.5; KCl, 0.1; Ca(NO_3_)_2_, 0.01 and yeast extracts 0.2. For routine culturing, the strain was cultivated in BSM (of pH 2.4) supplemented with 10 g/L S^0^ as an electron donor, at 45°C and 180 rpm.

### Bioleaching of metals from RM

A flask experiment was performed to investigate the effect of the S^0^/RM mass ratio on the rates of RM dealkalization and recovery of metals from RM. Sterile BSM (100 mL; pH 2.4) in Erlenmeyer flasks was supplemented with 0, 0.6, 1.2, and 1.8 g S^0^, and inoculated with *Sb. thermosulfidooxidans* to an initial cell density of 1 × 10^8^ cells/mL. The flasks were incubated in a shaking incubator at 45°C and 180 rpm. When the solution pH decreased to ~1.2 after 7 days of S^0^ bio-oxidation, 0.6 g of RM was added to each assay (giving the following S^0^/RM final mass ratios: 0:1, 1:1, 2:1, and 3:1) and bioleaching of metals from RM commenced. Each assay was performed in triplicate, and solution samples were regularly withdrawn every 1–4 days from the assays for kinetic measurements (for more detail see Analytical methods below). The losses due to water evaporation during the bioleaching process were compensated with deionized water, while the losses due to sampling were compensated with a fresh sterile medium.

**Figure 1:**
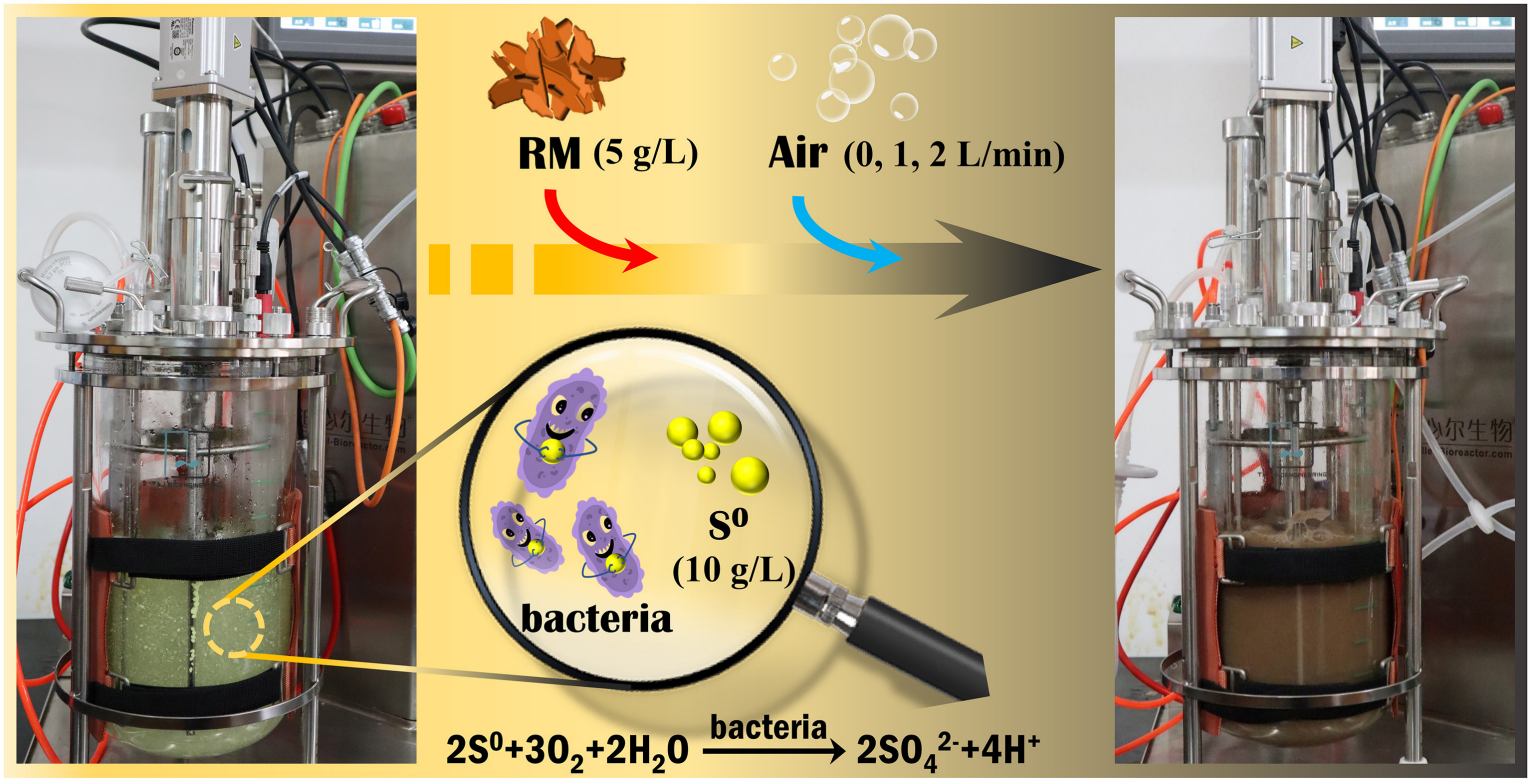
Experimental setup for bioleaching of valuable metals from RM by *Sb. thermosulfidooxidans* in a 5-L STR.

To improve the metal extraction from RM, another experiment was carried out in a 5 L stirred-tank reactor (STR) containing 2.5 L of sterilized BSM (pH 2.5) and 25 g S^0^ (i.e., 10 g/L). The STR was inoculated with *Sb. thermosulfidooxidans* to an initial cell density of 1 × 10^8^ cells/mL. The process parameters were set to 45°C and 140 rpm, controlled by the STR operation system (Fermac 200 modules equipped with Fermac 231 agitation module, all Electrolab, UK) (Figure 1). A sterilized industry standard Pt100 sensor was used for temperature control in the STR. Sterile air was supplied to the vessel through a 0.22 μm filter membrane. After the solution pH decreased to ~1.2, 12.5 g of RM was added (i.e., the final S^0^/RM mass ratio was 2:1). To investigate the effect of aeration on the rate of metal bioleaching from RM, biotic assays were performed with the aeration rates of 0, 1, and 2 L/min, together with an abiotic control with 1 L air/min. The STR experimental setup used in this study is shown in Figure 1.

### Analytical methods

The pH values were measured by using a Thermo Scientific Orion DUAL STARTM pH/ISE meter with a pH meter, and Eh values by a platinum (Pt) electrode with saturated calomel electrode (Hg/Hg_2_Cl_2_) as a reference electrode, with the Eh values normalized to a standard hydrogen electrode (SHE). Planktonic cell counts were monitored using a bacterial counting plate (HD-852) and a light microscope (Olympus BX43) in a phase contrast mode (400× magnification). Prior to the below analyses, the samples were filtered through 0.22 μm membranes (Millipore, Germany). The concentration of ${\text{SO}}_{4}^{2-}$ was determined by the barium sulfate turbidimetric colorimetric method (Tabatabai, 1974). The concentrations of dissolved Na, K, Ca Al, Si, Ti, and Fe were determined by inductively coupled plasma optical emission spectrometry (ICP-OES; FMX 26, SPECTROBLUE, Germany). The concentrations of Ce, Sc, and Y were measured by inductively coupled plasma-mass spectrometry (ICP-MS; 7700x, USA).

The surface morphology and elemental composition of solid residues were analyzed by a field-emission scanning electron microscope (FE-SEM; TESCAN MIRA3, Czech Republic), coupled with an energy-dispersive spectrophotometer (EDS; Hitachi S-4800, Japan). The Raman analysis was conducted on a laser Raman spectrometer (Jobin Yvon LabRam-010, France). The solid mineralogy was determined using X-ray diffraction (XRD; D/Max 2500, Japan). The Al and Si species were analyzed by X-ray photoelectron spectroscopy (XPS; K-Alpha+, Thermo Scientific, UK) equipped with an Al Kα X-ray source (15 KeV), with the voltage and current on X-ray of 12 kV and 6 mA, respectively. The distribution of Na in the solid residues was determined by scanning transmission X-ray microscopy (STXM) based on the dual-energy contrast imaging method at beamline BL08U1A of Shanghai Synchrotron Radiation Facility (SSRF), Shanghai, China.

## Results and discussion

### Effect of S^0^/RM mass ratio on bioleaching of metals from RM

**Figure 2:**
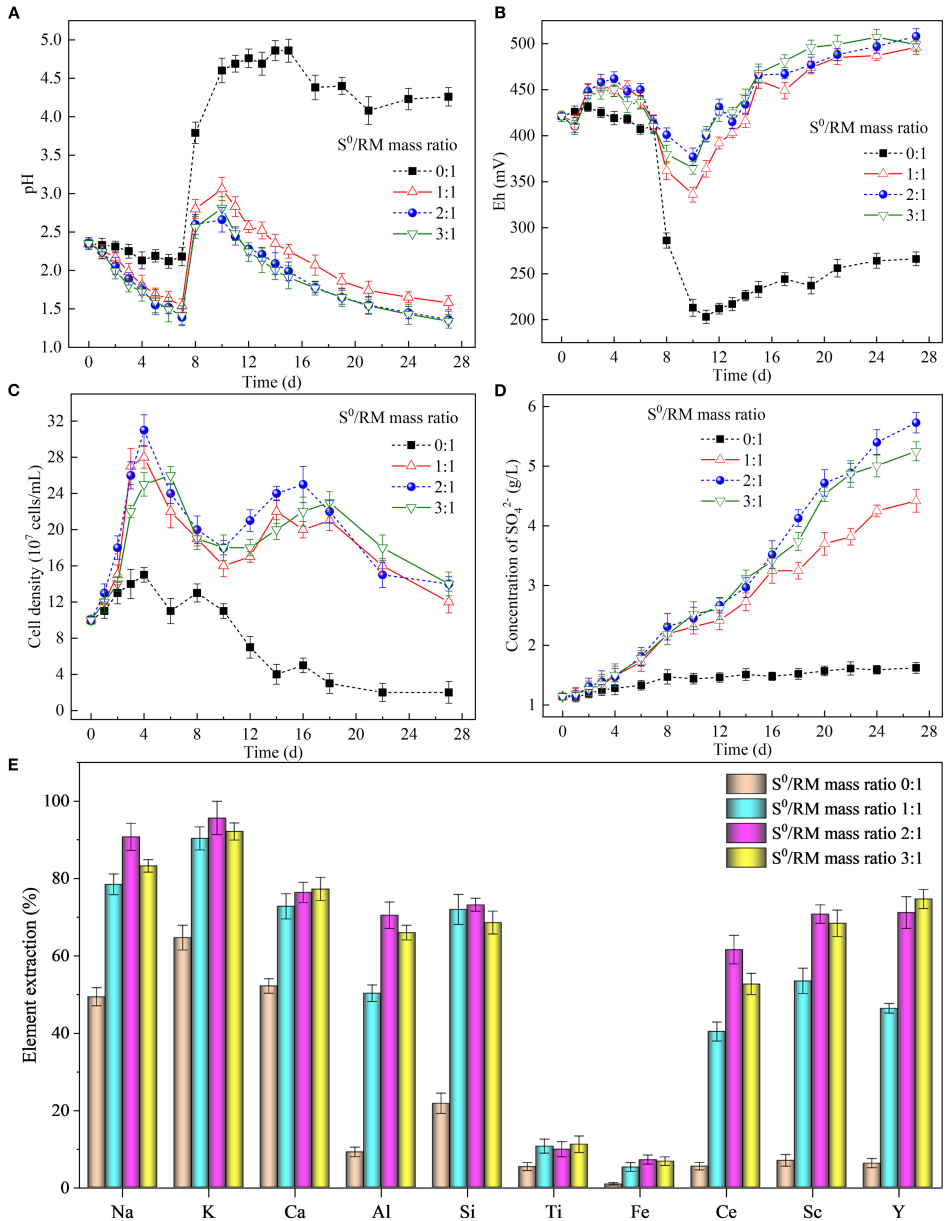
Effects of the S^0^/RM mass ratio on RM bioleaching process kinetics. Changes in **(A)** pH, **(B)** Eh, **(C)** planktonic cell counts, **(D)** concentration of ${\text{SO}}_{4}^{2-}$ during the bioleaching processes under S^0^/RM mass ratios of 0:1, 1:1, 2:1, and 3:1, and **(E)** percentages of elements extracted from RM into solution after 27 days of RM bioleaching.

As shown in Figure 2A, the pH in all bioleaching assays, except the assays without S^0^, decreased gradually in the first 7 days of cultivation due to acid production from S^0^ bio-oxidation by *Sb. thermosulfidooxidans*, with oxygen serving as electron acceptor. The bacterially catalyzed S^0^ oxidation process is described by Equations (1)–(3), where Equations (1) and (2) are half-reactions, and Equation (3) shows the full redox reaction (Vera et al., 2013). The supply of RM on day 7 resulted in a sharp increase in pH, due to a consumption of biogenic H_2_SO_4_ on neutralization of the alkaline components (particularly Na- and Ca-bearing phases) present in RM. The elevated pH caused by the addition of RM reduced the growth rate of the bacteria (Figure 2C). The acid generated *via* S^0^ bio-oxidation in assays containing S^0^ secured lower pH values that were more favorable for the growth and metabolism of *Sb. thermosulfidooxidans*. The highest pH was observed 3 days after RM addition (on day 10), reaching 3.0, 2.5, and 2.6 in the assays with the S^0^/RM ratios of 1:1, 2:1, and 3:1, respectively. These values were visibly lower than the pH determined in the bioleaching assay without S^0^ (reaching 4.9; Figure 2A). From day 10, a continuous decrease in pH occurred in all bioleaching assays that contained S^0^, due to the generation of biogenic H_2_SO_4_. The final pH in the assay with the S^0^/RM mass ratio of 1:1 was 1.6, while a lower pH value (~1.4) was determined in assays with greater S^0^/RM mass ratios. It was concluded that up to the S^0^/RM mass ratio of 2:1, the extent of the pH decrease was proportional to the amount of S^0^ present in the assay.


(1) $$\begin{array}{r} S^{0}+4H_{2}O\overset{\text{microbial}}{\to }SO_{4}{}^{2-}+8H^{+}+6e^{-} \end{array}$$

(2) $$\begin{array}{r} \begin{array}{c} 1.5O_{2}+6H^{+}+6e^{-}\overset{\text{microbial}}{\to }3H_{2}O \end{array} \end{array}$$

(3) $$\begin{array}{r} \begin{array}{c} S^{0}+1.5O_{2}+H_{2}O\overset{\text{microbial}}{\to }SO_{4}{}^{2-}+2H^{+} \end{array} \end{array}$$

The addition of RM on day 7 caused a rapid drop in Eh in the assays (Figure 2B), likely due to the surface of RM being negatively charged within the experimental pH range (Liu et al., 2013). In all assays, Eh reached a minimum value on day 10, after which a gradual increase was observed throughout the time course of the experiment likely due to the release of Fe during RM dissolution (Supplementary Figure S3). This was attributed to the mobilization of metal cations (predominantly Al^3+^, Ca^2+^, K^+^, and Na^+^) *via* acidic dissolution of RM mediated by S^0^ bio-oxidation, and potential bio-oxidation of the solubilized metals (particularly Fe).

Figure 2C shows that the planktonic cell density increased in the first 4 days of the experiment, reaching the maximum of ~31 × 10^7^ cells/mL. A decrease in cell density followed, likely due to inhibition of the bacterial growth by low pH (~1.4) (Golovacheva and Karavaiko, 1978; Christel et al., 2018). Coincidentally, the sudden increase in pH caused by the addition of RM also negatively affected the bacterial growth (as well as S^0^ oxidation, as shown in Figure 2D). However, this short-term effect was followed by an increase in cell density (and ${\text{SO}}_{4}^{2-}$ concentration in Figure 2D) from day 12 to 17 (when the pH ranged between 2.6 and 1.8), followed by another decrease toward the end of the experiment.

The extraction of elements from RM (Figure 2E) increased with the increasing S^0^/RM mass ratio from 1:1 to 2:1, while a further increase to 3:1 did not, in general, result in elevated element solubilization. The maximum extraction of Na, K, Al, Si, Fe, Ce, and Sc (reaching 91, 96, 70, 73, 7, 62, and 71%, respectively) was achieved with the S^0^/RM mass ratio of 2:1, while the maximum extraction yields of Ca, Ti and Y (77, 11, and 75%, respectively) occurred at the S^0^/RM ratio of 3:1. The dissolution of less stable aluminosilicates in RM such as kaolinite [Al_2_Si_2_O_5_(OH)_4_] (Equation 4), cancrinite [Na_6_(Al_6_Si_6_O_24_)(CaCO_3_)•2H_2_O] (Equation 5), katoite [Ca_3_Al_2_(SiO_4_)(OH)_8_] (Equation 6), and resulted in an efficient release of Na, Ca, and Al (Pepper et al., 2016; Zhou et al., 2021). The accumulation of H_4_SiO_4_ due to the dissolution of silicate phases in an acidic solution can contribute to the formation of silicate gel, lowering the recovery of Si (López-García et al., 2017; Khairul et al., 2019). The Ca-bearing phases in RM, such as calcite [CaCO_3_] and hydrogarnet [Ca_3_Al_2_(SiO_4_)_x_(OH)_12−4x_], are generally easily solubilized under acidic conditions. However, Ca dissolution from RM in this study was low due to the formation of insoluble calcium sulfates, as confirmed by the XRD analysis (**Figure 5**). The very low extraction of Ti and Fe (both <15%) was likely due to the elements occurring mainly as perovskite [CaTiO_3_], kassite [CaTi_2_O_4_(OH)_2_], and hematite [Fe_2_O_3_], all of which are relatively stable under the experiment pH (Sidhu et al., 1981; Salmimies et al., 2012). Also, proportions of Ce and Sc in RM were present in forms difficult to dissolve (such as CeO_2_ and Fe(III) oxide lattice), which likely lowered the element dissolution rates (Li et al., 2013; Borra et al., 2015).


(4) $$\begin{array}{r} Al_{2}Si_{2}O_{5}{\left(OH\right)}_{4}+6H^{+}\to 2Al^{3+}+2H_{4}SiO_{4}+H_{2}O \end{array}$$

(5) $$\begin{array}{l} Na_{6}\left(Al_{6}Si_{6}O_{24}\right)\left(CaCO_{3}\right)•2H_{2}O+26H^{+}\to 6Na^{+}+\\ \;Ca^{2+}+6Al^{3+}+6H_{4}SiO_{4}+CO_{2}+3H_{2}O \end{array}$$

(6) $$\begin{array}{l} Ca_{3}Al_{2}\left(SiO_{4}\right){\left(OH\right)}_{8}+12H^{+}\to 3Ca^{2+}+2Al^{3+}+\;\\ \;H_{4}SiO_{4}+8H_{2}O \end{array}$$

To summarize, the changes in solution pH and Eh caused by the dissolution of alkaline RM and S^0^ bio-oxidation by *Sb*. *thermosulfidooxidans* affected the bacterial growth and S^0^ oxidation rates, thus affecting also the recovery rates of elements from RM.

### Solid residues after bioleaching of metals from RM

**Figure 3:**
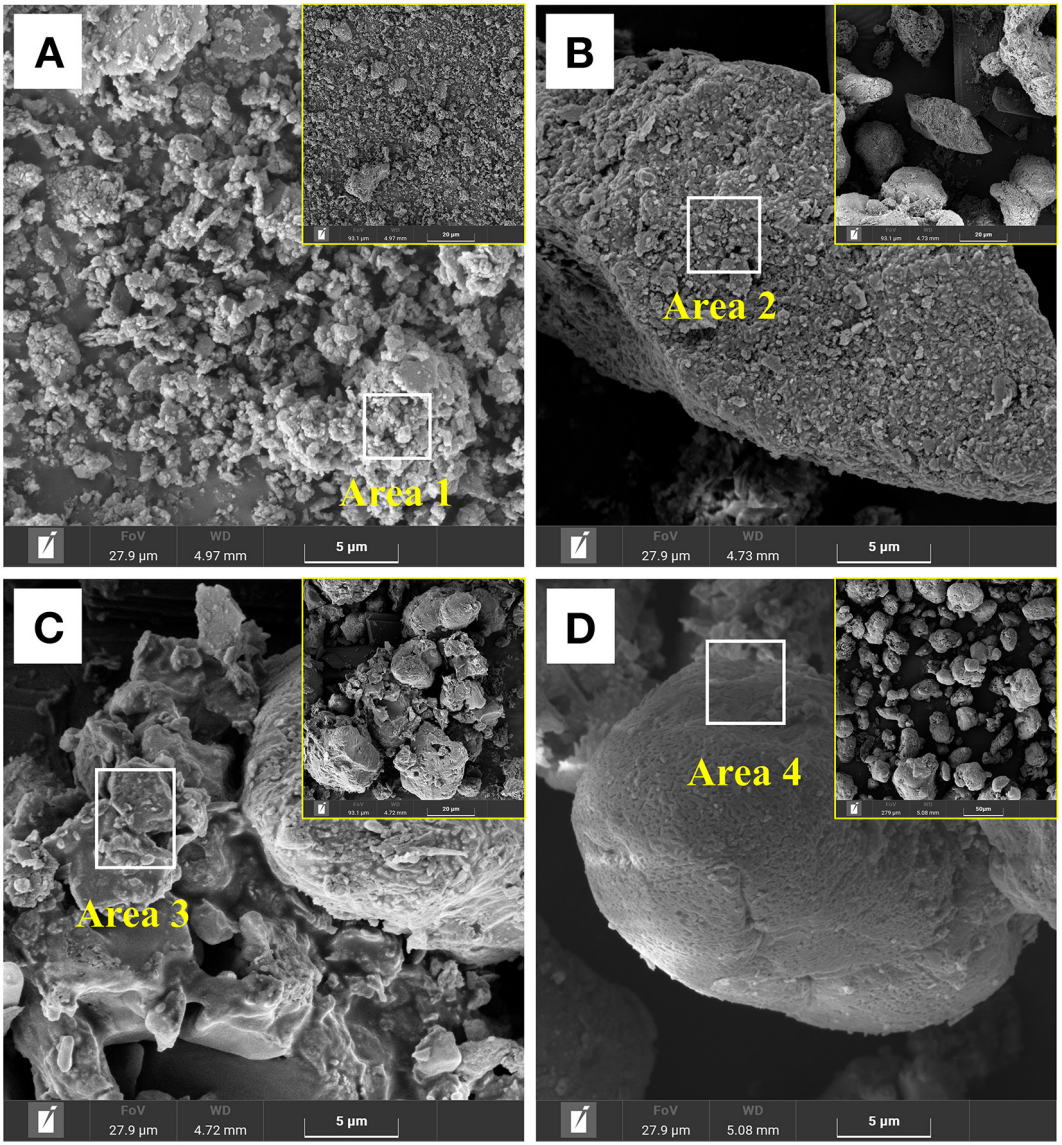
SEM images of solid residues after bioleaching of metals from RM by *Sb. thermosulfidooxidans*. Bioleaching was conducted for 27 days in shake flasks with the S^0^/RM mass ratio of **(A)** 0:1, **(B)** 1:1, **(C)** 2:1, and **(D)** 3:1. Areas 1, 2, 3, and 4 are the EDS analysis positions.

The surface morphology of the leached residues is shown in Figure 3. The residues collected after 27 days of RM bioleaching from the assay without S^0^ had an irregular flake-like structure (Figure 3A) similar to that of original RM (Supplementary Figure S1), indicating that *Sb*. *thermosulfidooxidans* could not mediate the dissolution of RM in the absence of S^0^. In the assays with the S^0^/RM mass ratio of 1:1, large amounts of the fine sheets corresponding to RM were densely covering the surface of S^0^ (Figure 3B). In contrast, no morphology typical for RM was detected in the bioleaching residues from the assays with the S^0^/RM mass ratio of 2:1, and the surface of the S^0^ present was severely corroded by bacterial cells (Figure 3C). In the assays with the S^0^/RM mass ratio of 3:1, the RM morphology was again mostly absent, while the surface of S^0^ was relatively intact and covered with a thin amorphous layer comprising mainly O, Al, Si, Fe, and K (Figure 3D).

**Figure 4:**
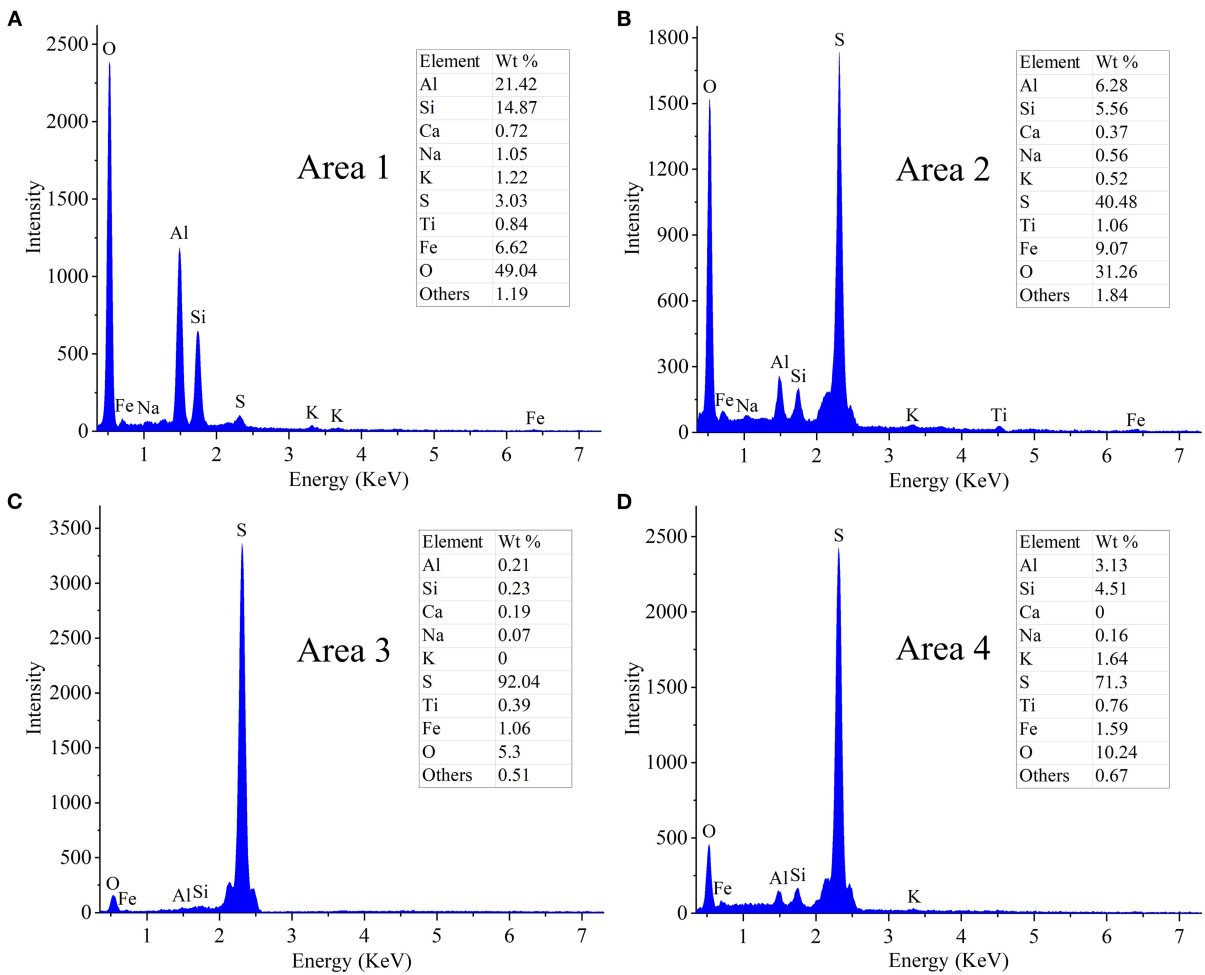
EDS spectra of solid residues after bioleaching of metals from RM. Bioleaching was conducted for 27 days in shake flasks with the S^0^/RM mass ratio of **(A)** 0:1, **(B)** 1:1, **(C)** 2:1, and **(D)** 3:1. EDS spectra in **(A–D)** correspond to Areas 1, 2, 3, and 4 marked in Figure 3 by white rectangles, respectively.

The results of the EDS analysis of elemental composition of the solid residues after RM bioleaching are shown in Figure 4. The residues from the assays without S^0^ comprised (in wt.%) 49 O, 21.4 Al, 14.8 Si, 6.6 Fe, 1.2 K, and 1.1 Na (Figure 4A). In the residues from the bioleaching assays with the S^0^/RM mass ratio of 2:1, the contents of O, Al, Si, and Fe decreased to 5.3, 0.2, 0.2, and 1.1%, respectively, and Na and K almost disappeared (Figure 4C). With a further increase in the S^0^/RM mass ratio (to 3:1), greater proportions of Al and Si remained in the solid phase (Figure 4D), indicating the dissolution of RM was partially inhibited. The adsorption of cells onto S^0^ particles is the key process for bacteria growth and H_2_SO_4_ production (Nguyen and Lee, 2015). The high solid _(${\text{S}}_{\text{+}}^{\text{0}}\text{RM}$)_/liquid ratio (1 g/42 mL) caused by addition of too much S^0^ (S^0^/RM mass ratio 3:1) resulted in a decreased effective contact between the cells and S^0^ particles, and thus inhibition of RM dissolution.

**Figure 5:**
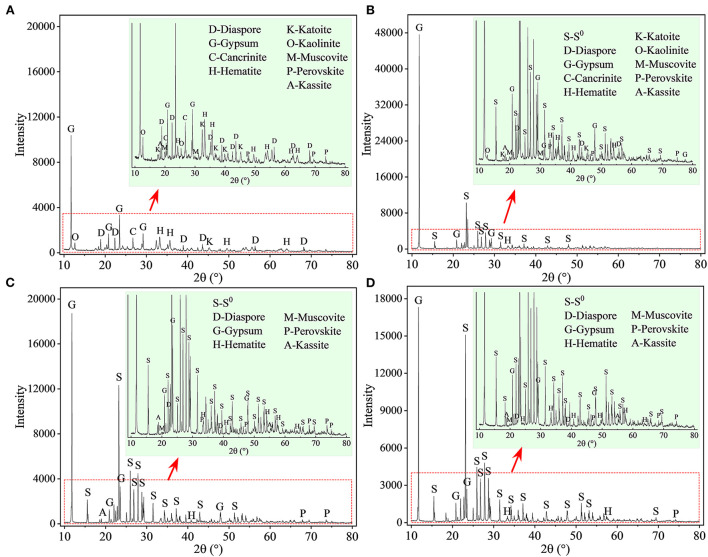
XRD patterns of solid residues after bioleaching of metals from RM. Bioleaching was conducted for 27 days in shake flasks with the S^0^/RM mass ratio of **(A)** 0:1, **(B)** 1:1, **(C)** 2:1, and **(D)** 3:1. The zoom image represents the enlarged area delineated by the red rectangle.

The XRD patterns of the leached residues are shown in Figure 5. Most of the phase peaks typical for RM (i.e., diaspore [β-AlO(OH)], cancrinite [Na_6_(Al_6_Si_6_O_24_)(CaCO_3_)·2H_2_O], hematite [Fe_2_O_3_], katoite [Ca_3_Al_2_(SiO_4_)(OH)_8_], kaolinite [Al_2_Si_2_O_5_(OH)_4_], muscovite [KAl_2_Si_3_AlO_10_(OH)_2_], perovskite [CaTiO_3_], and kassite [CaTi_2_O_4_(OH)_2_]) were detected in the residues from the assays without S^0^ after 27 days of bioleaching (Figure 5A). With increases in the mass ratio of S^0^ to RM (Figures 5B–D), the intensities of these peaks decreased. The diffraction peaks for cancrinite, katoite, and kaolinite were not detected in the spectra of the residues from assays with the S^0^/RM ≥ 2:1, besides small amounts of hematite, diaspore, perovskite, and muscovite, which were relatively stable under acidic conditions (Agatzini-Leonardou et al., 2008; Zhang et al., 2022). Newly formed gypsum (CaSO_4_·2H_2_O) and the remaining S^0^ became the main phases in the bioleached residues (Figures 5C,D).

**Figure 6:**
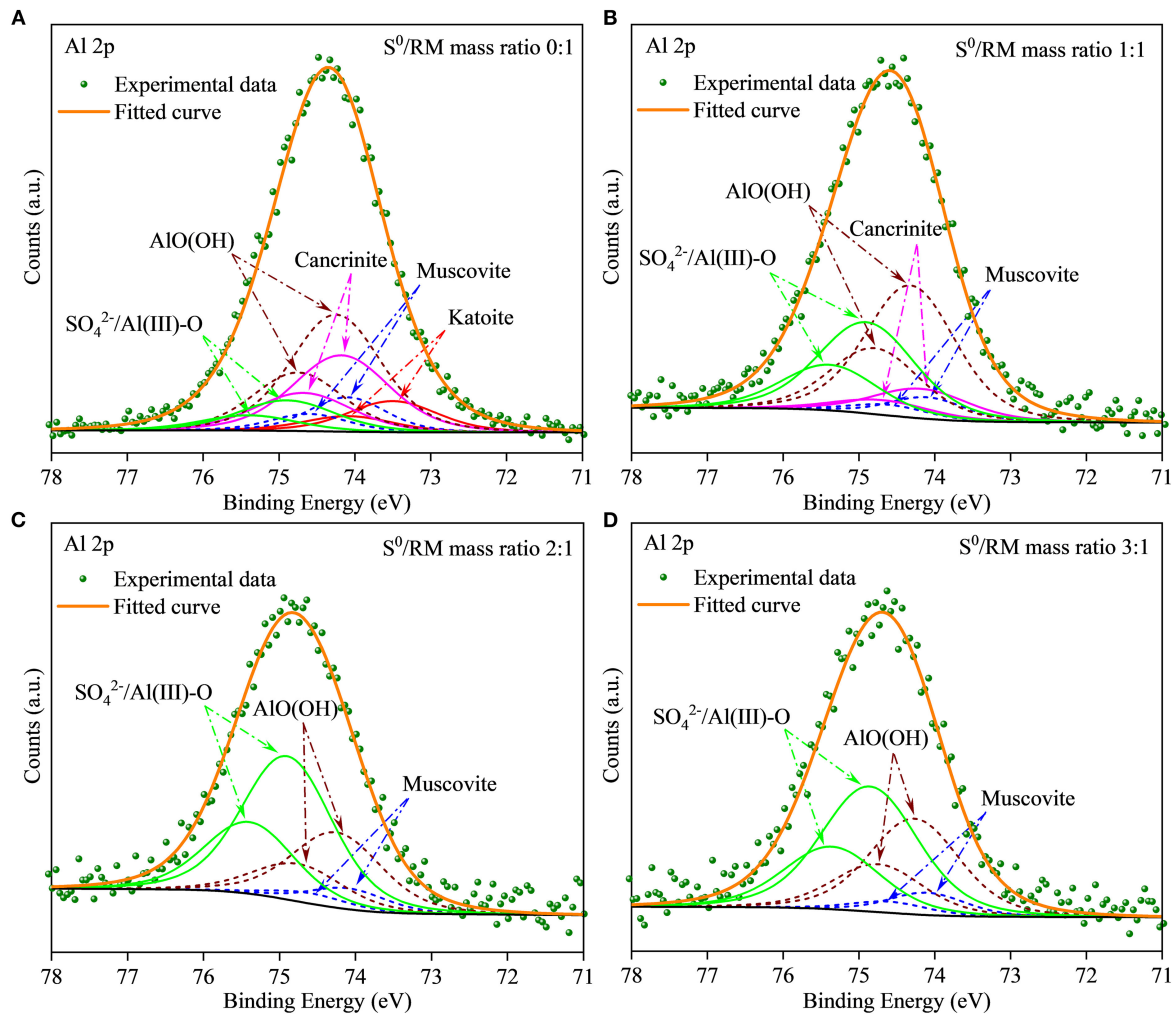
XPS spectra of Al in solid RM residues after bioleaching by *Sb. thermosulfidooxidans*. Bioleaching was conducted for 27 days in shake flasks with the S^0^/RM mass ratio of **(A)** 0:1, **(B)** 1:1, **(C)** 2:1, and **(D)** 3:1.

Furthermore, to investigate the evolution of Al species during RM bioleaching, the XPS analysis was performed, and the results are shown in Figure 6 (with the fitted parameters listed in Supplementary Table S1). The Al species in the residues from the assays without S^0^ (Figure 6A) composed mainly of β-AlO(OH) (74.22 eV), cancrinite (74.17 eV), muscovite (74.10 eV), and katoite (73.60 eV), with a lower proportion of newly formed ${\text{SO}}_{4}^{2-}$/Al(III)-O (75.0 eV) (Sherwood, 1998). These results were consistent with the XRD results shown in Figure 5. When the mass ratio of S^0^/RM increased to 1:1, katoite was not detected in the residues, the contents of muscovite and cancrinite decreased, and that of ${\text{SO}}_{4}^{2-}$/Al(III)-O increased (Figure 6B; Supplementary Table S1). In the assays with the S^0^/RM mass ratio of 2:1, the Al species in the residue comprised mainly ${\text{SO}}_{4}^{2-}$/Al(III)-O and β-AlO(OH), with a lower proportion of muscovite (Figure 6C). Similar results were obtained for the S^0^/RM of 3:1 (Supplementary Table S1).

It can be concluded that bio-oxidation of S^0^ by *Sb. thermosulfidooxidans* efficiently promoted the dissolution of RM, proving the feasibility of the biotechnology for larger-scale recovery of metals from the bauxite residue. However, the findings also show that an excessive S^0^ content (S^0^/RM mass ratio 3:1 in this study) resulted in a decrease in the recovery of metals from RM. The results emphasize the importance of optimizing the process parameters, and indicate that the most favorable S^0^/RM mass ratio for efficient bioleaching of metals from RM was 2:1. This was supported by the high dissolution rates of Na^+^ and K^+^ (> 90% in the assays with the S^0^/RM mass ratio of 2:1) showing that sufficient S^0^ oxidation by *Sb. thermosulfidooxidans* was achieved for effective dealkalization of RM.

### Effect of aeration on RM bioleaching in a 5-L STR

During bioleaching processes based on acid dissolution, chemolithotrophic bacteria oxidize reduced sulfur compounds (such as S^0^) and transfer electrons through the electron transport chain to oxygen (Bonnefoy and Holmes, 2012). Adequate supply of oxygen is therefore crucial to promote bio-oxidation of S^0^ and bioleaching of metals (Lizama, 2001; Witne and Phillips, 2001; De Kock et al., 2004; Mazuelos et al., 2017).

**Figure 7:**
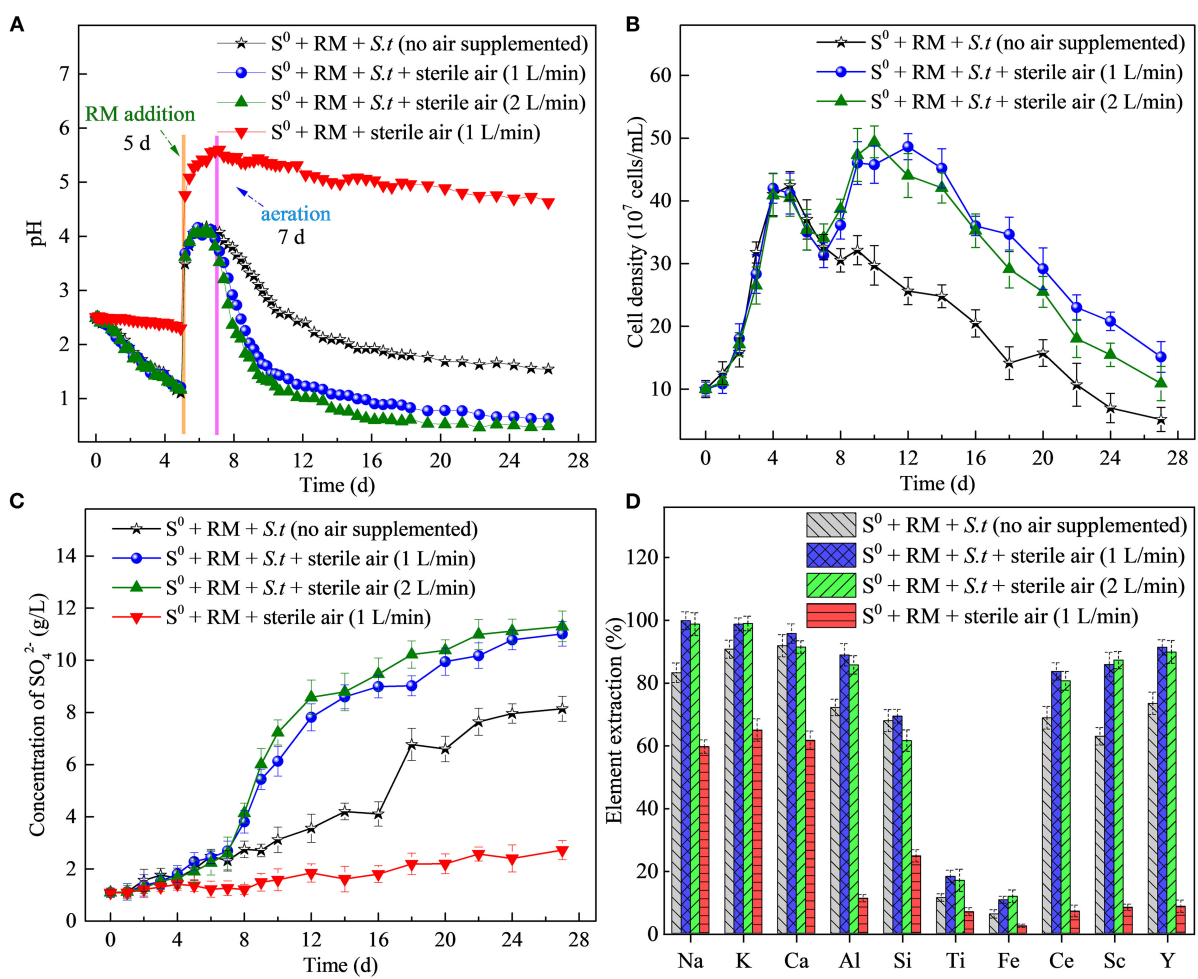
Effects of the aeration rate on RM bioleaching process kinetics. Changes in **(A)** pH, **(B)** planktonic cell counts, **(C)** concentration of ${\text{SO}}_{4}^{2-}$, and **(D)** percentages of elements extracted from RM. Bioleaching by *Sb. thermosulfidooxidans* (*S.t*.) was conducted for 27 days in a 5-L STR with the S^0^/RM mass ratio of 2:1.

Similarly to the above described flask experiment (shown in Figure 2A), a rapid increase in solution pH (Figure 7A) occurred in STR after RM addition (on day 5), negatively affecting the rates of bacterial growth (Figure 7B) and sulfur oxidation (Figure 7C). To mitigate the negative effects of the elevated pH, sterile air (1 and 2 L/min) was supplied to the STRs from day 7 to promote S^0^ bio-oxidation. Following the commencement of aeration, a rapid decrease in pH was observed in both biotic assays (with 2 L air/min slightly faster than with 1 L air/min), compared to the slower pH decrease without air supply. The above results clearly indicate that aeration had a profound effect on the S^0^ bio-oxidation rate. Also, the bacterial growth was in accordance with the pH changes observed, the planktonic cell densities reaching the maxima of 4.86 × 10^8^ and 4.94 × 10^8^ cells/mL in the biotic assays with 1 (on day 12) and 2 L air/min (on day 10) respectively (Figure 7B).

Sulfate concentrations in biotic assays (reaching 11 and 11.3 g/L at the aeration rate of 1 and 2 L/min, respectively) were visibly greater than those in the biotic assays without aeration (8.1 g/L) and abiotic control with 1 L air/min (2.5 g/L), confirming the effect of aeration on S^0^ bio-oxidation (Figure 7C). The highest extraction rates of Al (89%), Ce (84%), Sc (87%), and Y (91%) were achieved in the biotic assays with aeration (1 or 2 L/min), with the values higher than those in the biotic assay without air supply and abiotic assays (Figure 7D). In the biotic assay with the aeration rate of 1 L/min, the extraction rates of Ce and Y increased rapidly from day 8 to 20 and reached a maximum on day 24, followed by a slight decrease (Supplementary Figure S4). The decrease in the extraction rate of Ca on day 22 could be attributed to the formation of gypsum. On the other hand, the extraction rate of Ti increased (reaching 18%) compared to the flask bioleaching experiment, which can be ascribed to an increased rate of dissolution of Ti-bearing phases in RM due to a higher concentration of H^+^ generated from more efficient S^0^ bio-oxidation (Agatzini-Leonardou et al., 2008). Based on the solubilization rates of Na and K, the dealkalization rate of RM reached nearly 100% in the biotic assay due to aeration.

**Figure 8:**
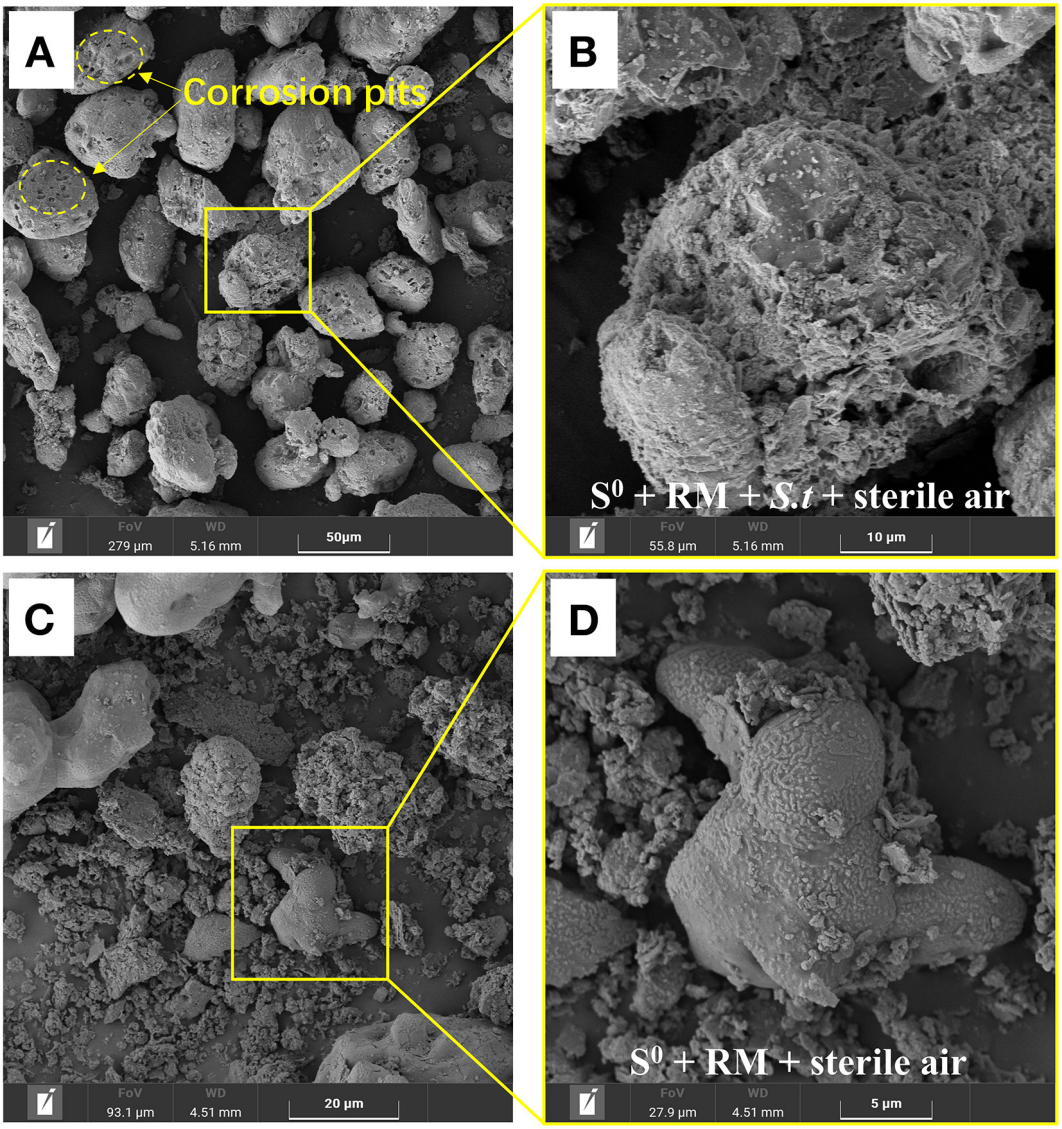
SEM images of the solid residues after leaching of metals from RM. **(A,B)** Biotic assays with aeration, **(C,D)** abiotic assays with aeration, where **(B,D)** represent enlarged areas marked in **(A,C)**, respectively. Bioleaching was conducted for 27 days in a 5-L STR with the S^0^/RM mass ratio of 2:1 and aeration rate of 1 L/min.

**Figure 9:**
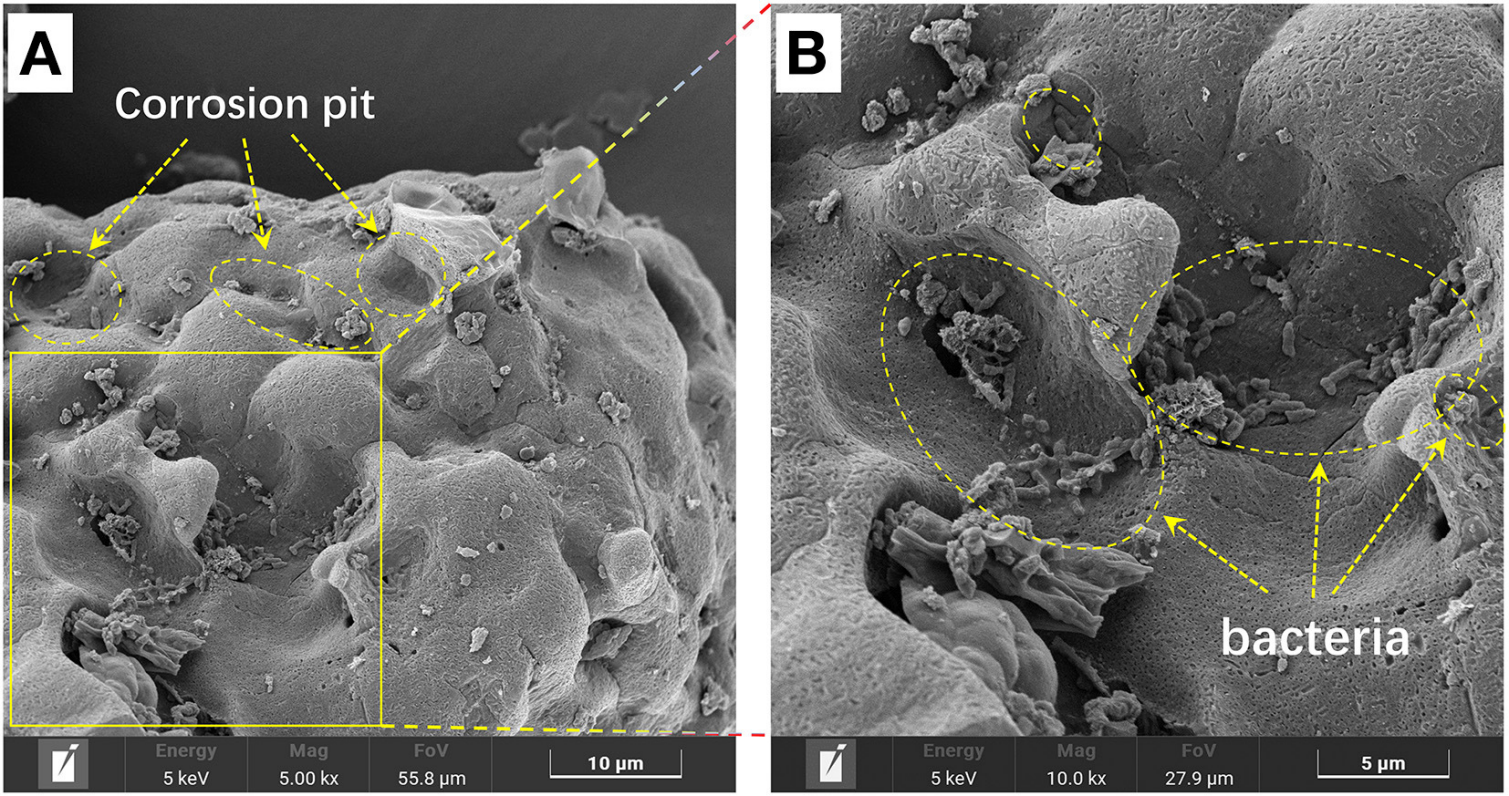
SEM image of the bio-oxidized residue, where **(B)** represents the enlarged area that is delineated in **(A)**.

After 27 days of bioleaching, the irregularly sheet-like structures corresponding to RM disappeared in residues from the biotic assays with aeration (1 L/min) (Figures 8A,B). The surface of S^0^ was greatly corroded (i.e., showing numerous corrosion pits; Figure 8B) due to sulfur bio-oxidation by *Sb. thermosulfidooxidans*, and many bacterial cells was found attached in the corrosion pits (Figure 9). These findings further indicate that the adsorption of bacteria onto the S^0^ particles was an important part of the S^0^ oxidation process. In comparison, copious amounts of RM were detected in the abiotic assays with the same aeration rate, and the surface of residual S^0^ was smooth and intact (Figures 8C,D). A comparison with the SEM images of residues from shake flasks without air supply (shown in Figure 3C) clearly indicates that aeration promoted the bacterial S^0^ oxidation process that mediates metal leaching and RM dealkalization.

**Figure 10:**
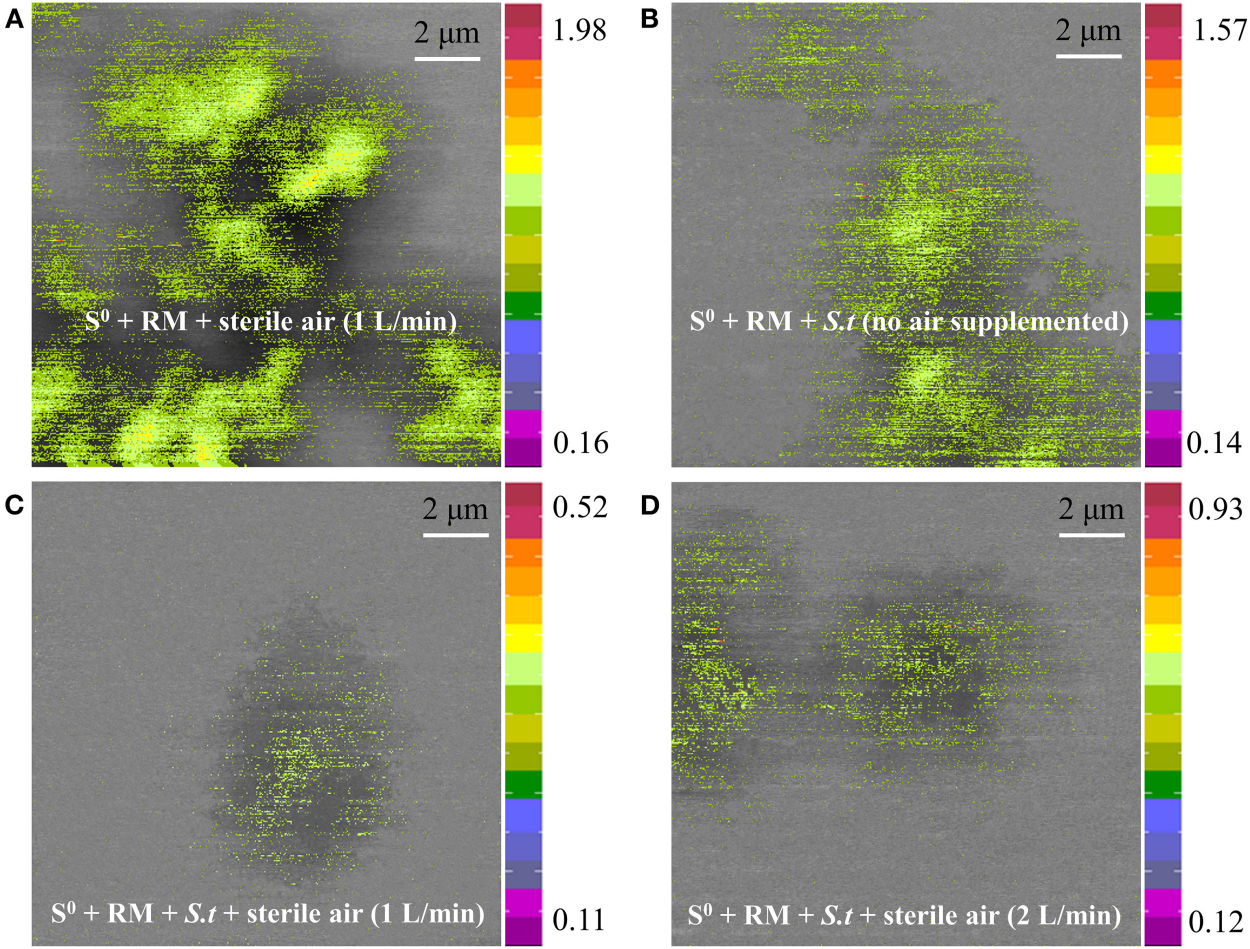
Sodium distribution on the surface of solid residues after RM bioleaching, analyzed by STXM. Leaching was conducted for 27 days in a 5-L STR with the S^0^/RM mass ratio of 2:1. Residues from the following assays were analyzed: **(A)** S^0^ + RM + 1 L air/min; **(B)** S^0^ + RM + *Sb. thermosulfidooxidans* (*S.t*) + 0 L air/min; **(C)** S^0^ + RM + *S.t* + 1 L air/min; **(D)** S^0^ + RM + *S.t* + 2 L air/min. The numbers next to the color bars indicate the maximal and minimal contents of Na in each mapping (unit: 1 × 10^−5^ g/cm^2^).

Furthermore, the distribution of Na on the residues after RM bioleaching was analyzed by STXM (Figure 10), to determine the degree of RM dealkalization. The following Na contents on the surface of the residues were determined: 1.98 × 10^−4^ to 0.16 × 10^−5^ g/cm^2^ in abiotic assays (Figure 10A), 1.57 × 10^−5^ to 0.14 × 10^−5^ g/cm^2^ in biotic assays without air supply (Figure 10B), 0.52 × 10^−5^ to 0.11 × 10^−5^ and 0.93 × 10^−5^-0.12 × 10^−5^ g/cm^2^ in biotic assays with 1 (Figure 10C) and 2 L/min (Figure 10D) aeration rates, respectively. The lowest Na content was detected in the residue from the biotic assays with 1 L air/min, indicating this aeration rate was most suitable for metal dissolution and RM dealkalization.

**Figure 11:**
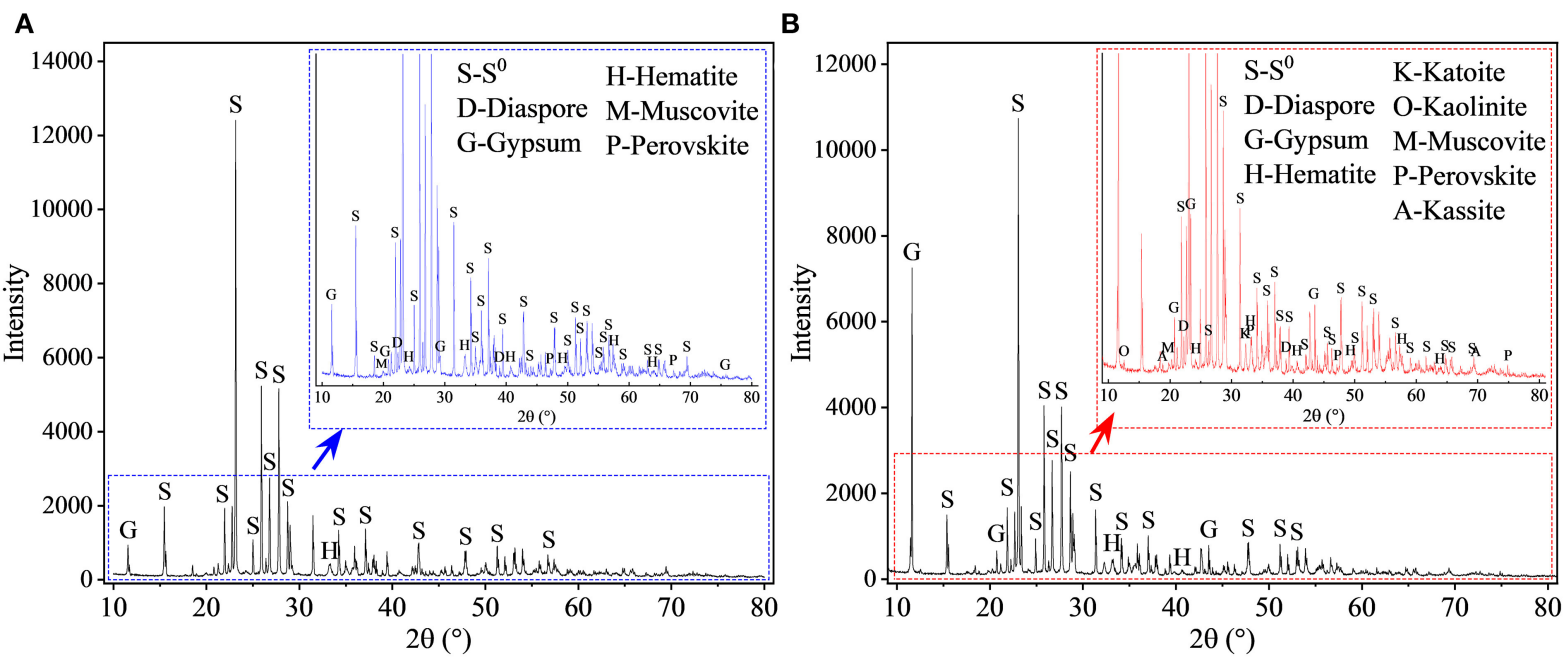
XRD patterns of solid residues after leaching of metals from RM. Leaching was conducted for 27 days in a 5-L STR with the S^0^/RM mass ratio of 2:1. Residues from the following assays were analyzed: **(A)** S^0^ + RM + *Sb. thermosulfidooxidans* + 1 L air/min; **(B)** abiotic control (S^0^ + RM + 1 L air/min). The zoom image represents the enlarged area delineated by the blue or red rectangle.

**Figure 12:**
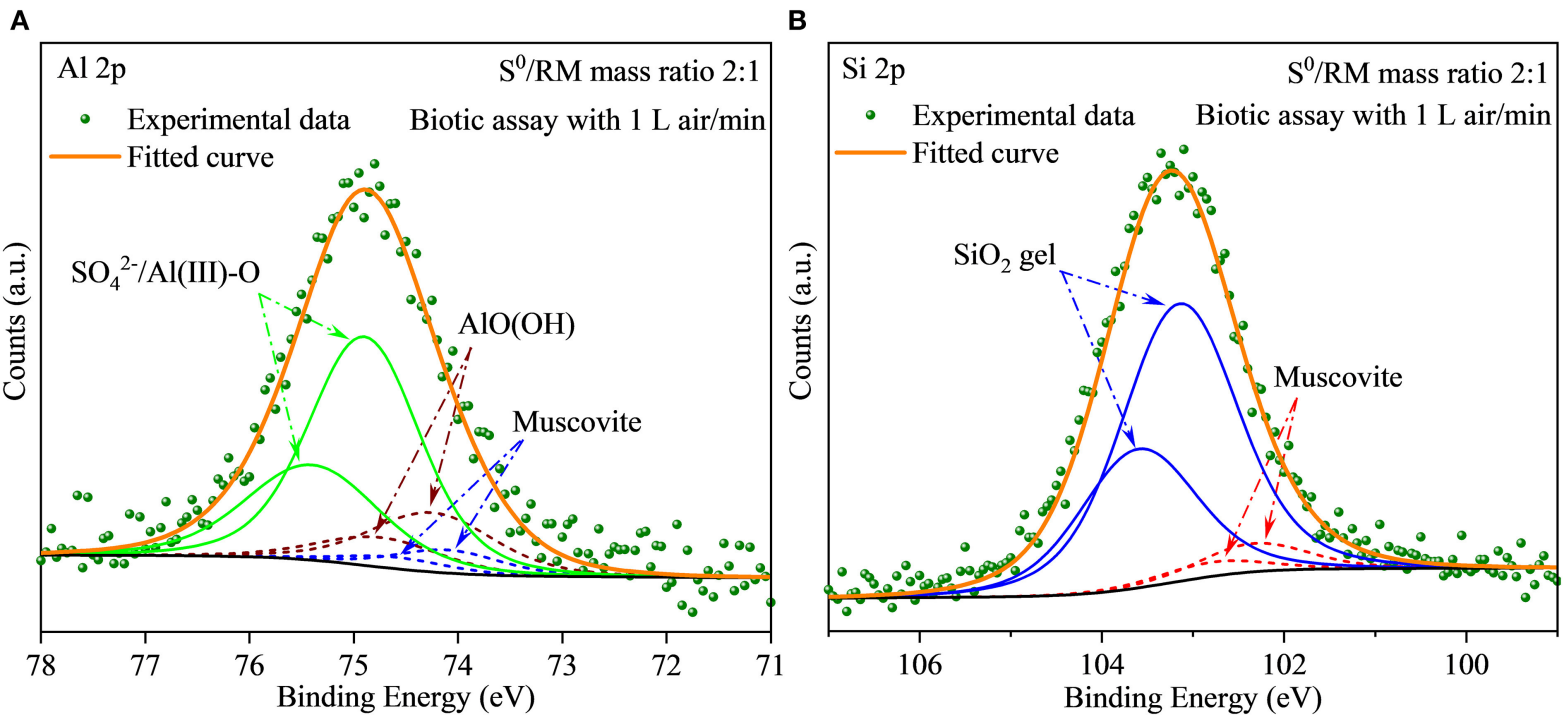
**(A,B)** XPS spectra of Al and Si in the solid residues after bioleaching of metals from RM. The bioleaching was conducted for 27 days in a 5-L reactor with the S^0^/RM mass ratio of 2:1.

The XRD results (Figure 11) show that after 27 days of leaching, most RM components disappeared in the solid residues from the biotic assays with 1 L air/min, except for some stable phases, e.g., hematite, diaspore, muscovite, gypsum, and perovskite (Figure 11A). Alkaline minerals (katoite, kaolinite, and kassite) were detected, besides the above mineral phases, in the solid residues from abiotic assays (Figure 11B). The abiotic residues had a similar mineral composition to the original RM, which often comprises hematite, kaolinite, gibbsite, calcite, quartz, and diaspore (Wang and Liu, 2012). Similarly to the above-described flask experiment (shown in Figure 6C), the surface Al species of the residue collected from the biotic assay with 1 L air/min were mainly composed of ${\text{SO}}_{4}^{2-}$/Al(III)-O and AlO(OH) with minor amounts of muscovite (Figure 12A), while Si was mainly present as muscovite and SiO_2_ gel (Figure 12B). The above indicates that most of the aluminosilicates in RM dissolved during the bioleaching process, and different Al/Si-bearing compounds were formed. These findings further support the importance of the role of microbial catalysis of S^0^ oxidation that mediates RM dissolution and dealkalization.

## Conclusions

In this study, the use of S^0^ oxidation by the moderately thermophilic bacterium *Sb. thermosulfidooxidans* was proven feasible to efficiently dealkalize red mud and achieve high recovery rates of valuable metals (i.e., Al, Ce, and Y) from this amassing industrial waste. The experimental results emphasize the importance of optimization of the process parameters, to promote S^0^ bio-oxidation and RM dissolution. Biogenic H_2_SO_4_ mediates RM dissolution and extraction of metals from RM, rates of which in this study increased with an increase in S^0^:RM mass ratio (up to 2:1). Aeration can enhance the S^0^ bio-oxidation efficiency and thus further improve the recovery of metals and RM dealkalization. In summary, the S^0^:RM mass ratio of 2:1 and aeration of 1 L/min were determined as the optimal conditions for the bioprocess, with the leaching rates of Al, Ce, and Y reaching 89, 84, and 91%, respectively, and the rate of RM dealkalization reaching ~100%, determined by the extraction rate of Na. Most of the aluminosilicate minerals were dissolved during the bioleaching process, except for a few stable phases such as hematite, perovskite, diaspore, and muscovite. These findings provide an insight that could help design cost-effective ways for recovering metals from RM, together with RM dealkalization. Further research could focus on increasing the production of biogenic H_2_SO_4_, lowering the aeration rate, and reducing the production of liquid waste (e.g., by media recirculation). Additionally, we suggest that by-products from gas and/or coal desulfurization processes could be used as the sources of S^0^ for the bacteria in large-scale industrial applications, which would greatly reduce the process operational costs.

## Data availability statement

The original contributions presented in the study are included in the article/Supplementary material, further inquiries can be directed to the corresponding author.

## Author contributions

D-rZ: conceptualization, investigation, formal analysis, data curation, and writing–original draft. H-rC: investigation and data curation. J-lX: funding, supervision, conceptualization, methodology, and writing–review and editing. Z-yN: resource and formal analysis. R-YZ and EP: writing–review and editing. All authors read and agreed to the published version of the manuscript.

## Funding

This work was supported by the Funds for International Cooperation and Exchange of the National Natural Science Foundation of China (No. 51861135305) and the Open Fund of Shanghai Synchrotron Radiation Facility (2022-SSRF-PT-021591).

## Conflict of interest

The authors declare that the research was conducted in the absence of any commercial or financial relationships that could be construed as a potential conflict of interest.

## Publisher's note

All claims expressed in this article are solely those of the authors and do not necessarily represent those of their affiliated organizations, or those of the publisher, the editors and the reviewers. Any product that may be evaluated in this article, or claim that may be made by its manufacturer, is not guaranteed or endorsed by the publisher.
