# Supplementary material for: Efficient dealkalization of red mud and recovery of valuable metals by a sulfur-oxidizing bacterium
Source: Front Microbiol. 2022 Aug 29;13:973568. doi: 10.3389/fmicb.2022.973568 (PMC9465049; doi:10.3389/fmicb.2022.973568)
Supplement: Supplementary file 1 [file Data_Sheet_1.docx]

*Supporting Information for*

**Efficient** **dealkalization of red mud and recovery of valuable metals by a sulfur-oxidizing bacterium**

Duo-rui Zhang ^1,2^, Hong-rui Chen ^1,2^, Jin-lan Xia ^1,2*^, Zhen-yuan Nie ^1^

Rui-Yong Zhang ^1,2,3,4^, Eva Pakostova ^5^

^1^School of Minerals Processing and Bioengineering, Central South University, Changsha 410083, China

^2^Key Lab of Biometallurgy of Ministry of Education of China, Central South University, Changsha 410083, China

^3^Key Laboratory of Marine Environmental Corrosion and Bio-fouling, Institute of Oceanology, Chinese Academy of Sciences, 7 Nanhai Road, Qingdao 266071, China

^4^Open Studio for Marine Corrosion and Protection, Pilot National Laboratory for Marine Science and Technology (Qingdao), 1 Wenhai Road, Qingdao 266237, China

^5^Centre for Sport, Exercise and Life Sciences, Coventry University, Coventry CV1 5FB, UK

*Correspondence to be addressed: Jin-lan Xia, E-mail: [jlxia@csu.edu.cn](mailto:jlxia@csu.edu.cn)


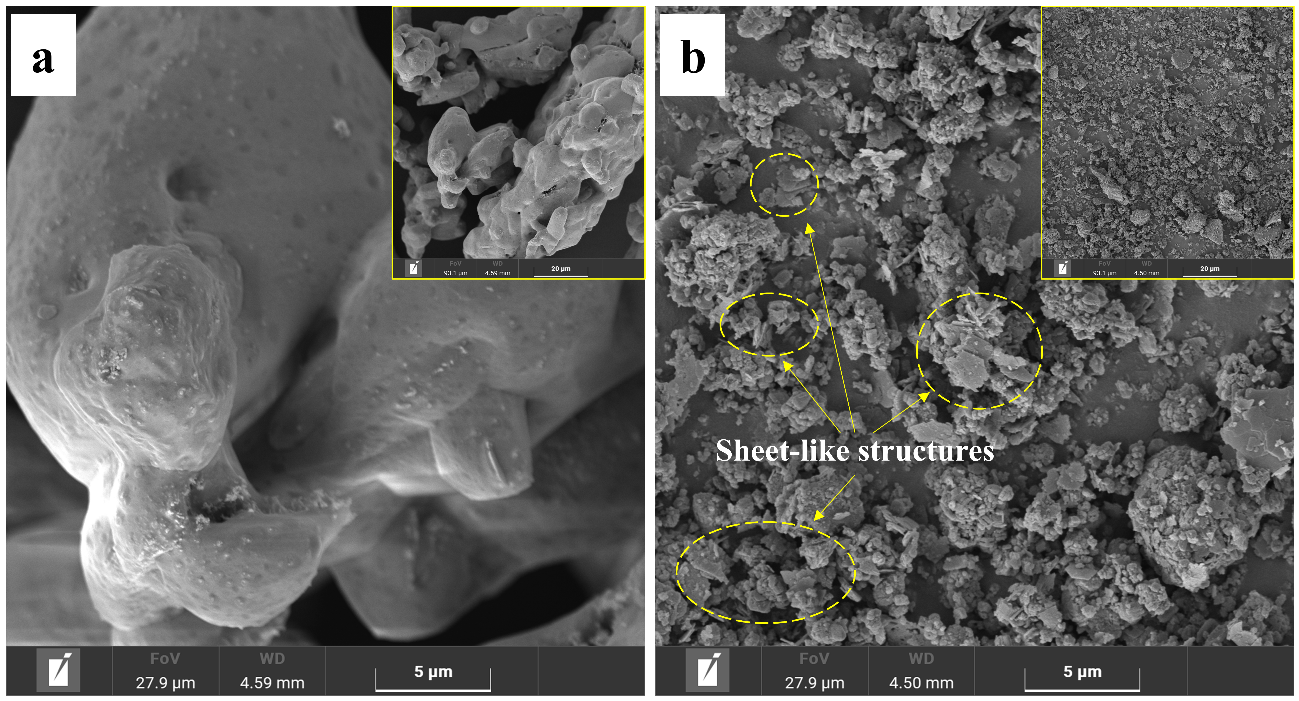


**Fig. S1.** SEM images of intact (a) elemental sulfur (S^0^) and (b) red mud (RM).

**Fig. S2**. XRD pattern of the original RM.

**Fig. S3.** Change in the concentration of Fe released from RM during the bioleaching processes. Bioleaching was conducted in shake flasks with the S^0^/RM mass ratio of 0:1, 1:1, 2:1, and 3:1.

**Fig. S4**. Changes in the extraction rates of Al, Ce, Y, Na, K, and Ca at different times of bioleaching in a 5-L STR with the S^0^/RM mass ratio of 2:1 and aeration rate of 1 L/min.

**Table S1** **Peak parameters for Al 2*P* spectra and proportions of Al species on the surface of solid residues after RM bioleaching**. Bioleaching was conducted for 14 days in shake flasks with the S^0^/RM mass ratios of (**a**) 0:1, (**b**) 1:1, (**c**) 2:1, and (**d**) 3:1.

| State | Katoite | Muscovite | Cancrinite | *β*-AlO(OH) | SO_4_^2-^/Al(III)-O |
| --- | --- | --- | --- | --- | --- |
| BE (eV) | 73.60 ± 0.1 | 74.10 ± 0.1 | 74.17 ± 0.2 | 74.22 ± 0.2 | 74.90 ± 0.1 |
| FWHM (eV) | 1.40 | 1.20 | 1.50 | 1.40 | 1.50 |
| S^0^/RM | (at. %) | (at. %) | (at. %) | (at. %) | (at. %) |
| 0:1 | 10.66 | 11.08 | 28.01 | 40.05 | 10.20 |
| 1:1 | - | 8.20 | 11.32 | 45.17 | 35.31 |
| 2:1 | - | 7.18 | - | 32.02 | 60.80 |
| 3:1 | - | 7.89 | - | 38.26 | 53.85 |
